# The changing landscape of training in microscopy facilities: Reflections on seven decades of research user support at the University of Sydney

**DOI:** 10.1111/jmi.70121

**Published:** 2026-05-28

**Authors:** Eleanor Kable, Matthew Foley, Vijay Bhatia, Filip Braet

**Affiliations:** ^1^ Sydney Microscopy and Microanalysis The University of Sydney New South Wales Australia; ^2^ Australian Centre for Microscopy and Microanalysis The University of Sydney Sydney New South Wales Australia; ^3^ School of Medical Sciences (Molecular Biomedicine) The University of Sydney New South Wales Australia

**Keywords:** core facilities, facility management, microscopy user challenges, online training tools, platform scientists, research training

## Abstract

This thematic contribution highlights the critical role of established professional training regimes in microscopy and microanalysis in supporting high‐quality research. As microscopy technologies continue to evolve in complexity, the risk of mishandling and data misinterpretation by inexperienced researchers increases significantly. To address this challenge, the authors underscore the importance of expert training and ongoing support provided by platform scientists—specialist microscopists embedded within centralised research infrastructure facilities. These experts play a vital role in equipping researchers with the necessary skills to use advanced instruments effectively and interpret data accurately. Collaboration between platform scientists and researchers is not only beneficial but essential for maximising the capabilities of modern microscopy. By fostering such partnerships and investing in structured training solutions, institutions can ensure that scientific discoveries are grounded in reliable, high‐quality imaging data, advancing the broader goals of research excellence. At the microscopy core facility in Sydney, we have found that no single training method can fully substitute the comprehensive researcher experience required to become an expert microscopy scientist. Therefore, we suggest that a hybrid approach—combining multiple training tools with regular researcher check‐ins throughout the research project lifecycle—is the most effective solution.

## LAY DESCRIPTION

- This thematic contribution, prepared for the festschrift honouring Dr. Guy Cox former Deputy Director of Sydney Microscopy & Microanalysis highlights the importance of established training regimes in microscopy and microanalysis in the pursuit of high‐quality research.
- The authors emphasise the importance of collaboration between platform scientistsnamely, expert microscopists who provide first‐class trainingand researchers, particularly within the context of centralised facilities.
- Good training practices in centralised research infrastructure facilities are essential to fully realising the potential of microscopy and, in turn, advancing scientific discovery.
- With the growing complexity of modern microscopes, there is an increasing need for expert training support to mitigate the substantial risk of incorrect use or misinterpretation of data by inexperienced microscopy users.

## 1 INTRODUCTION

The University of Sydney has a proud legacy in microscopy, beginning with the establishment of the Electron Microscope Unit (EMU) in 1958—the first centralised open‐access microscopy facility at any Australian university.^1^ The EMU was rebranded as Sydney Microscopy & Microanalysis (SMM) in 2012 to better reflect the unit's broader focus on all forms of microscopy. SMM is now supported by a team of professional and academic staff from diverse training backgrounds, providing research services at six major microscopy nodes across the university (Figure 1). Each of these nodes has been tailored to support the typical local microscopy and microanalysis needs within research precincts that heavily rely on microscopy equipment (e.g., physical, biological, engineering, and natural sciences). Importantly though is to keep the connectivity of these nodes as this enables knowledge transfer of commonality across these areas. This is achieved by the staff regularly coming together and belonging to the one overarching team.

**FIGURE 1:**
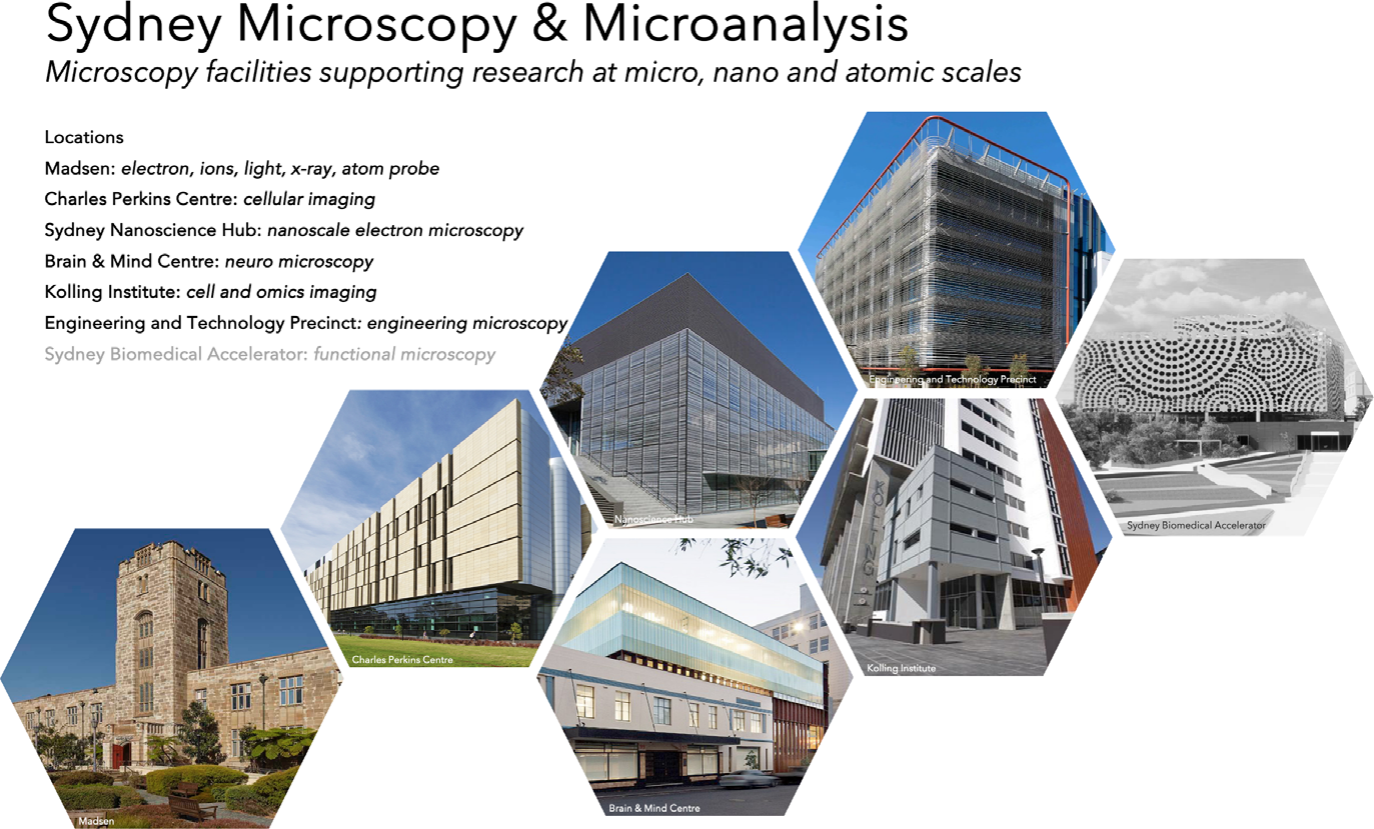
Sydney Microscopy & Microanalysis (SMM) operates at five locations (nodes) on the main campus, with a linked node at Royal North Shore Hospital (RNHS): (1) Madsen Building: 21 microscopy platforms and 47 support tools (i.e. ancillary tools for sample preparation or to process data collected from instruments); (2) Charles Perkins Centre: 14 instruments and 7 support tools; (3) Brain and Mind Centre: 8 instruments and 4 support tools; (4) Sydney Nanoscience Hub: 2 instruments and 3 support tools; (5) Engineering Microscopy: 5 instruments and 2 support tools; and (6) Kolling Institute (linked node within RNHS): 2 instruments. Note: SMM is currently in advanced planning for a new node in a biomedical research building under construction (monochrome image), focused on functional microscopy to support its drug discovery and structural biology research programs.

Running multidisciplinary microscopy facilities comes with challenges,^2^ which are compounded when operating across multiple microscopy sites delivering distinct imaging capabilities (i.e., electron‐, light‐, ion‐, X‐ray microscopy). One of these challenges is establishing a training regime and workflow that fits everyone's needs. Decades of experience taught us that there is the ongoing need for fine‐tuning the training regime which we call the ‘new user experience’ (see *below*). This thematic contribution precisely discusses the training aspect and how we have to adapt to the ever‐changing researcher experience in an ever‐evolving microscopy landscape.^3^ Herein, we share our experiences and present a robust well‐crafted cycle of a user training program that supports the needs of researchers—whether they are doctoral candidates, postdoctoral fellows, early‐career researchers, or established scientists.

## 2 RESEARCHER EXPERIENCE

In today's complex research environment—where publishing a single paper often requires access to a wide array of advanced technologies and highly specialised equipment—expert staff (platform scientists) within core facilities have become indispensable.^4^, ^5^ No single researcher can master all technologies, let alone afford the full suite of instrumentation. This reality, however, presents a significant challenge for microscopy facilities: are we training researchers to become competent end users, or are we cultivating the next generation of microscopy specialists? In truth, the answer lies somewhere in between.

Training has been an integral part of the SMM's activities almost since day one—specifically since 1960, when it was known as EMU.^6^ As Ratinac and Simpson wrote in their chapter on training at Sydney's microscopy facility: ‘Of course, any shared facility has a clear responsibility to provide at least basic user training—no staff member wants to see expensive instruments damaged by inexperienced users. Nor is it in the long‐term interest of a centralised facility to allow users to produce poor‐quality data due to a lack of fundamental understanding of the instruments and techniques involved. While these are valid and necessary reasons for user training, EMU's commitment to delivering high‐quality instruction goes well beyond the standard expectations of good practice in centralised facility operations’. There is no better way to succinctly summarise the importance of appropriate user training support in any facility. From this emerged our graphical representation of the new user experience^7^—a cycle of steps that guides research users from A to Z in their microscopy endeavours (Figure 2). Variants and fine‐tuning based on emerging challenges have been discussed and implemented in our facility (see next section)—for example, to address issues related to big data.^4^

**FIGURE 2:**
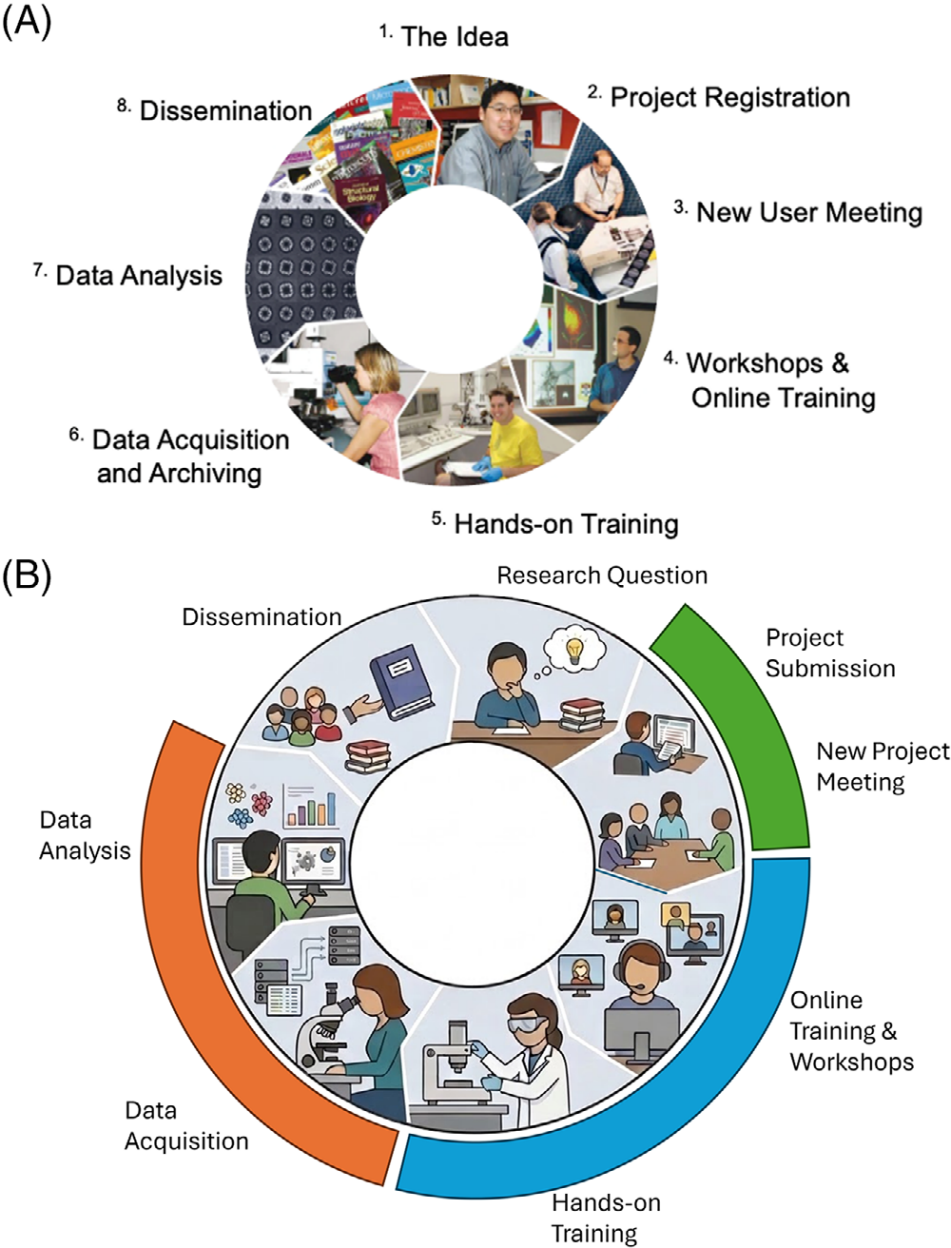
Diagram depicting the user experience at Sydney Microscopy & Microanalysis (SMM) that has stood the test of time and forms the basis for our researcher training. After the idea is generated by the scientist and the new project request is submitted through our online portal, the researcher is then guided and supported through SMM's researcher experience, which consists of workshops‐, and/or short introduction presentations, and/or online learning, and finally hands‐on training. Once training is completed and the researcher is assessed on their competencies, they can independently operate the equipment they were trained on and acquire data. The final steps include data analysis and dissemination of the results (e.g., theses, publications, posters, conferences, etc.). Support for data analysis is a growing challenge.^4^ Our facilities provide basic support, and for more complex analyses, we connect researchers with specialists within our network. (A) One of the first diagram versions, dating back to 2006.^7^ (B) A reimagined version highlights the three identified areas of refinement we have been working on since 2020: (1) *The Submission & Review Phase (Green)*, focusing on project submission and resource allocation; (2) *The Training Phase (Blue)*, emphasising the need for appropriate learning modalities and tiered proficiency; and (3) *The Data Collection, Analysis & Curation Phase (Orange)*, addressing increasingly complex microscopy technologies and techniques, along with the growing challenges in data analysis and specialist support.

The research user experience at SMM is designed as a continuous cycle of expert‐guided support throughout the microscopy workflow. Platform scientists lead researchers through each stage of their research, starting with planning (via the new project meeting), followed by hands‐on training, data acquisition and processing, and continuing with guidance on data presentation and the accurate formulation of materials and methods. This model not only serves as a best practice for training researchers but also ensures their work meets the highest scientific standards, with sustained support and access to research data. This researcher experience at SMM has stood the test of time. However, with the ever‐changing landscape of microscopy, the emergence of innovative technologies, and the increasing complexity brought by multimodal imaging, the way we approach and implement the various steps needed to be adjusted. It is in this phase that the importance of technically skilled and professionally trained expert staff or platform scientists becomes pivotal.^5^ Their adaptability to new technologies, years of experience in microscopy facilities, strong connections with developers, and active engagement with industry and vendors enable them to train and offer research users contemporary, timely solutions—ultimately paving the way for high‐quality, accelerated microscopy science.^8^, ^9^, ^10^

## 3 EMERGING CHALLENGES IN TRAINING AND ADAPTING USER SUPPORT SYSTEMS

Emerging challenges in training scientists in microscopy are multifaceted and highlight the urgent need for innovation in user support systems, as advancing technologies require more specialised knowledge and adaptable, user‐centred training approaches.^3^, ^11^ One of those main challenges in microscopy training is adapting to increasingly complex hybrid systems that integrate multimodal imaging capabilities. This is accompanied not only by the inherent complexities of sample preparation but also by the vast amount of data generated, which requires careful management.^12^ In the latter case, as microscopes become more automated, researchers risk becoming overly reliant on software that provides push button operation with ready‐to‐use data on the fly (e.g., for publication), which can lead to gaps in fundamental understanding of the underlying microscopy and increase the risk of data misrepresentation.^13^, ^14^ The trend to simplify microscopy operation by introducing the push button microscope is dangerous. Good fundamental knowledge of how the microscope works which is supported by expert platform scientists remains essential to ensure quality data is collected following best practise. On another front, given the international trend towards shortening university programs (e.g., Honours, MScR, PhD)^15^ and the prevailing ‘publish or perish’ mentality, our approach to the researcherexperience has had to evolve over the past decade.^3^

Since 2020, we have identified three major areas of change and improvement that can maintain the integrity of the user experience while addressing modern pressures:

1. **Operational efficiency and cross‐node mobility**: Addressing the strain of managing multiple specialised sites.
2. **Integration of digital and E‐learning tools with adaptation of training**: Moving towards a hybrid model that balances online theory with in‐person assessment, along with customising training for shortened university timelines and diverse user proficiency needs.
  The expected goals for the researcher to demonstrate at the in person assessment are provided at the beginning of the first training session (see Appendix for examples).
3. **Supporting data acquisition and analysis**: Optimising image collection processes before assisting in the teasing out of data from increasingly complex datasets and volumes.

In this paper, we predominantly show the successes that we have had in the first two areas, along with a partial success in the third. Data analysis is an ongoing challenge that requires significant investment in staffing, hardware, and software resourcing. For further discussion on the importance of image data support, we recommend the dedicated paper written by one of this paper's authors.^4^

### 3.1 The challenge of onboarding of researchers and their unique research questions

As researchers enter the SMM environment, their onboarding journey is critical. Our microscopy facilities have expanded across six specialised nodes. To manage this growth, we have implemented a structured onboarding process to ensure consistent user training and safety engagement from the outset. Each of SMM's six nodes operates with unique microscopy and microanalysis technologies and sample workflows. Our facility management system plays a pivotal role in this process, as project details are reviewed upon submission and appropriate staff are allocated based on researcher needs. Following New Project Meetings, researchers are then directed to the relevant equipment within the facility. Within this decision‐making process is the consideration of resource availability as it relates to the research question to avoid oversubscription across equipment in areas where similar technique offerings are available across multiple locations.

As training requirements are highly section‐specific, where each section is primarily focussed on supporting a single microscopy technique, necessitating tailored programs that align with the instruments, research focus, and platform staff expertise in each node and section. The complexity of microscopy projects varies widely. Indeed, there is a world of difference, for example, between light optical experimentation in biomedical research and electron microscopy in supporting materials science. Therefore, general training and support must be customised not only to the research project but also to the specific instrument(s) involved, including location or node specific general induction training. This often means staff must design flexible and modular training pathways to suit different experimental workflows. Lately, we have moved heavily towards developing and continually revising relevant training materials, including manuals, interactive workshops, flash cards, and online training content (see below). This is undoubtedly a time‐saver for both staff and researchers.

By centralising project registration through a robust management system, we ensure that research questions are matched with the most appropriate equipment and node expertise before training begins. This proactive alignment prevents equipment oversubscription and allows for the design of modular, section‐specific training pathways that are strictly tailored to the researcher's immediate experimental goals. This structured onboarding transforms the potential bottleneck of diverse researcher needs into a streamlined workflow that maximises both instrument efficiency and data quality.

### 3.2 When ‘success’ strains the system

Terms like cross‐node mobility, lost productivity, and shifting workload frequently come up in staff meetings as key facility management operational challenges. With instruments spread across six nodes, staff and researchers often need to move between physical locations. This cross‐node mobility can lead to scheduling inefficiencies, duplication of training, and lost time, both for researchers and staff—reducing overall productivity. SMM, for example, has two nodes that provide transmission electron microscopy (TEM) services, two for scanning electron microscopy (SEM), and three nodes almost exclusively dedicated to optical microscopy (along with offering optical equipment in shared node spaces).

The rapid expansion of SMM and growth in instruments and researchers has significantly increased administrative responsibilities and training workload. Even though online interfaces add value and save time, we have found that the freed‐up capacity is quickly offset by the onboarding of more microscopy researchers. Coordinating training, maintaining usage records, managing access, and handling documentation now consume a substantial portion of staff time that could otherwise be devoted to scientific support. Along with that comes the tendency towards ‘too many (ad hoc and other) meetings’. While meetings are necessary for coordination, they often disrupt focused technical work and reduce the availability of our platform scientists for researchers’ engagement and instrument time, as well as limit staff development opportunities needed to acquire new expertise.

For this, we established strict new meeting guidelines for our staff (i.e., keep meetings to essential, group the meetings together both physically and in time where possible, and strictly adhere to allocated times). We have also, where practical, introduced protected time blocks for platform scientists to help balance coordination needs with uninterrupted technical work and staff development time. We encourage quick corridor or at‐the‐instrument conversations to check on researcher progress. This may sound simple, but it is important, as potential issues can be identified early, thereby reducing the risk of loss due to unknowingly doing something wrong. Our facility management system is instrumental in this, as it allows our staff to easily see who has booked instrument time. Finally, we have been consolidating our staff meetings into fewer, more targeted monthly or 6‐weekly section meetings (e.g., light optical, biological electron microscopy, etc.). This has allowed us not only to monitor researcher progress more effectively but also to engage in deeper discussions on technical and scientific challenges—saving time and helping us avoid the need to repeatedly ‘rediscover the tour’.

To mitigate the operational strain of multi‐node mobility and administrative growth, we implemented ‘protected time blocks’ for technical work and consolidated ad‐hoc staff interactions into targeted, discipline‐specific monthly meetings. By utilising our facility management system to monitor real‐time instrument bookings, staff can pivot from formal meetings to ‘at‐the‐instrument’ check‐ins, identifying potential technical issues before they result in poor researcher experiences. This shift from administrative coordination to proactive technical engagement ensures that facility growth does not come at the expense of platform scientist availability or staff professional development.

### 3.3 Maximising E‐learning and digital training platform success

In 2011, Microscopy Australia (MicroAu) launched a repository of online learning tools for microscopy—MyScope.^16^, ^17^ This collection of interactive modules, training texts, and quizzes has proved invaluable for microscopists around the world. The modules cover almost all conventional microscopies and are regularly updated, with new ones added as needed. It also includes topics about sample preparation, data management, and health and safety. In addition, in 2020, due to a reduction in face‐to‐face operations because of the pandemic, SMM staff prepared new and updated training material in the form of lectures, flashcards, online assessment and video material (e.g., YouTube, the CANVAS university learning management system, etc.). The new lectures and quizzes covered a sizable portion of theory that was previously being covered during legacy pre‐training face‐to‐face lectures and hands‐on training sessions. The assumption was that this would allow researchers and platform scientists to prepare themselves for a more streamlined experience when face‐to‐face training resumed.

Interestingly, rather than improving researchers' understanding of basic microscopy, post‐pandemic we have observed a decline in the average practical knowledge of researchers who exclusively complete the online modules. Although the modules are tailored for new microscopy users, the online quizzes are often brute forced by researchers interested in getting a pass mark, resulting in minimal self‐engagement. As a result, many researchers still require a considerable time investment with in‐person theory sessions before hands‐on training can begin. This observation aligns with broader educational research suggesting that e‐learning tools are most effective when integrated into a ‘blended’ model rather than serving as a standalone replacement for expert instruction.^18^ To mitigate the ‘brute‐forcing’ of online modules, we have followed this suggestion and transitioned to a blended competency model. While MyScope provides the theoretical framework, we have introduced a mandatory in‐person ‘Pre‐Flight’ check for techniques that require a researcher to show a pass‐mark for the online quiz—such as the ‘Foundations of Light Microscopy’ (FoLM) assessment or an in‐person SEM check. Before a researcher touches the instrument, a platform scientist conducts a 15‐min oral or practical quiz based on the online content. Internal feedback suggests this hybridity ensures that digital productivity gains are not lost to poor data quality or instrument downtime, as it forces a transition from passive consumption of digital content to active application of knowledge. This approach benefits both researchers and trainers by saving time and reducing workload, provided that the researchers microscopy knowledge is adequately assessed and supported in person by the platform scientist.

To prevent the ‘brute‐forcing’ of digital content, we have moved beyond simple pass‐fail online quizzes to a blended competency model that requires a mandatory in‐person ‘Pre‐Flight’ check. By integrating tools like MyScope and internal video resources with specialised assessments such as the ‘Foundations of Light Microscopy’ (FoLM), we ensure that theoretical knowledge is actively validated before hands‐on training commences. This hybrid approach protects staff time and instrument integrity while significantly increasing the ‘practical readiness’ of researchers in a time‐constrained academic environment.

### 3.4 Navigating shortened and diverse university curricula

With a growing number of researchers, clear communication about what SMM can offer within specific timeframes or under project‐based time constraints—and what researchers can expect—is vital. This includes realistic timelines, the depth of training, milestones, and researcher versus staff responsibilities—all of which help prevent misunderstandings and optimise both the use of microscopy time and the quality of microscopy data. As touched on before, considering the shortening of training programs, training needs differ significantly based on the researcher's project length and type. Short‐term researchers (e.g., Honours students) require rapid, task‐focused training, while long‐term researchers (e.g., PhD candidates) need deeper, ongoing skill development—adding to staff planning complexity. Recognising this researcher profile is important, as no one wants to invest time training a researcher as all‐round expert microscopist when the project duration is only a few months or, at most, a year. This has been a topic of debate in many meetings over the years, as we previously offered every single researcher the opportunity to receive training from basic to advanced microscopy techniques. For light microscopy, this requires gaining full knowledge of the principles of the light microscope, and likewise for electron optics in electron microscopy. This aspect is now largely captured by the online modules we offer and, if relevant to the researchers needs, followed up with face‐to‐face discussions (and assessment).

This means that the first question our staff ask themselves when reviewing the information submitted by users is: what is the required proficiency level—should the researcher be a ‘skilled professional microscopist’? Or is the researcher to be a novice with enough background to collect the desired data? Or perhaps, do they just need an image to help complement/complete their research study? After all, regular interaction (i.e., through check‐ins at the instrument) with our expert staff may help bridge the gap between each of these levels of training and support as the researcher progresses. It is often unclear from the research project description what level of competency researchers are expected to achieve. Should they become fully independent, or will they need a certain degree of support by staff? This highlights the importance of the first step in the researcher training process: the new researcher meeting. Calibrating training based on individual needs and future goals is essential, including tracking researcher progress towards becoming a skilled microscopist is one of our key responsibilities. Often, researchers thrive within SMM and develop into expert microscopy scientists. For us, this is a clear indication that we have successfully achieved our training mission.

By utilising the ‘New ProjectMeeting’ to calibrate training against project duration and desired proficiency, we ensure that staff effort is aligned with the researcher's actual needs. This tiered approach allows us to deliver rapid, task‐focused instruction for short‐term students while reserving deep, ongoing skill development for those aiming to become expert microscopists. Ultimately, tailoring training regimes at the local node level enables faster delivery and maintains high data standards, even within the constraints of shortening university curricula.

### 3.5 The challenge of doing everything

The challenge for a single researcher to master all the necessary techniques for a high‐impact publication is immense. In two recent papers involving SMM research—one from *Nature*,^19^ the other from *Neuropathologica* ^20^—there were a significant number of techniques and skills required. The *Nature* paper required skills ranging from animal handling and intricate tissue preparation to advanced correlative imaging and statistical analysis. At the same time, the *Neuropathologica* paper, while slightly less demanding, still required proficiency in human tissue handling, protein purification, advanced spectrometry, and complex data analysis. For a single individual, acquiring ‘moderate’ proficiency across all techniques for the *Nature* paper would take an estimated 2.5 to 3 years of dedicated training, and for the *Neuropathologica* paper, approximately 2 years. This timeframe is purely for skill acquisition and does not include the time needed for actual data collection, experimentation, or addressing the scientific questions themselves. This reality underscores the critical role of the hybrid training model offered by facilities like SMM. Instead of expecting one person to become an expert in every technique, the facility provides access to specialised equipment and, more importantly, to platform scientists. These experts serve as collaborators, guiding researchers and providing the technical proficiency needed for complex projects. This model allows researchers to focus on their core scientific questions while leveraging the deep technical knowledge of facility staff, making ambitious, multi‐disciplinary research projects feasible within realistic timeframes.

The traditional model of training every researcher to full independence is becoming unsustainable as high‐impact research now requires a level of technical mastery that would take years for a single individual to acquire. By transitioning to a collaborative model where platform scientists assist in high‐end data collection, we enable researchers to focus on their core scientific questions rather than the intricacies of instrument operation. This hybrid approach—balancing training for independence with expert‐led service—is the only viable way to deliver complex, multi‐modal research projects within the shortening timeframes of modern academic programs.

### 3.6 How to optimise support offerings on the fly

We have begun piloting minor adjustments aimed at optimising staff time and providing better support to researchers and their project. We found that group onboarding sessions improve initial training efficiency. They also provide microscopy researchers in training an opportunity to meet peers and exchange knowledge and ideas. Sometimes, our staff decide that it is faster and more efficient—especially for shorter projects where high‐end advanced microscopy (e.g., FIB‐SEM, cryogenic imaging) cannot be taught within the limited time window or microscopy is not a core part of the research work, that our staff, together with the researcher in attendance, operate the microscopes to facilitate the research. Although this is not our preferred model, staff have the discretion to make this call when deemed necessary. This frees up substantial imaging (and staff) time.

Finally, SMM categorises its researchers into four categories. Category 1 (Cat‐1) includes project registered researchers who are currently in training. Category 2 (Cat‐2) consists of intermediate researchers with 9‐to‐5 access, while Category 3 (Cat‐3) researchers have received additional training, demonstrated their competency, and are granted 24/7 access. Where possible, we further recognise advanced researchers as Category 4 (Cat‐4), staff‐acknowledged, fully skilled microscopists in a specific technology or platform. These Cat‐4 researchers are considered senior peers who can—if they wish contribute to mentorship and training support. Experimenting with moving more researchers from Cat‐2 to Cat‐3/Cat‐4 status is advisable. We consider our advanced researchers as peer microscopist colleagues, and we often see them go on to join (our own or other) microscopy facilities as platform scientists—highlighting the educational and training role that microscopy facilities provide!

By implementing a tiered classification system (Cat 1–4) alongside a staff‐led ‘Expert Operator’ model, we ensure that technical support is perfectly matched to both the complexity of the instrument and the timeline of the research project. This agile approach allows platform scientists to bypass lengthy training periods for high‐end modalities when a researcher's residency is too short to reach full competency, prioritising data delivery over unnecessary instruction. This hybrid framework maximises instrument efficiency and fosters a professional community where advanced ‘Cat‐4’ researchers act as peer‐mentors, ensuring the facility remains a hub for both high‐quality science and expert training.

## 4 CONCLUSION

We have identified and refined key steps within the training cycle as discussed above to arrive at a hybrid model that incorporates e‐tools available to us, including innovation in discipline‐specific (i.e., microscopy technique) training support. Our fine‐tuned training model was primarily driven by the ever‐changing landscape of microscopy demands and the shortening of higher research education programs, which has forced us to continuously adapt how we manage knowledge and train researchers within core research infrastructure facilities, maximising output with statistical rigor and high standards. To support this mission, we have explored various training tools over the years, including online educational resources, group‐based sessions, and face‐to‐face instruction.

In our Core Facility, we have found that no single method can fully replace the comprehensive researcher training experience, which typically involves a minimum of 4 to 10 h of hands‐on instruction—depending on the complexity of the microscopy technology (e.g., photons, electron, ions, X‐rays)—followed by several practice sessions with the platform scientist available for support. Additional hours are then required for data analysis, again varying with the complexity of the equipment and data involved. Our findings are in agreement with Imreh et al.^11^ who state ‘Two sessions of a few hours is very little time for researchers to learn to use the equipment with confidence, independently recognise and avoid the pitfalls of microscopy’. We propose that a hybrid approach—combining multiple training tools with regular researcher check‐ins throughout the research project lifecycle—is the most effective.

For emerging facilities, we recommend three actionable steps:

1. Implement mandatory online theory modules coupled with a face‐to‐face component with validation to preserve staff time for hands‐on ‘quality’ training.
2. Adopt a tiered researcher access system (Cat 1–4) to incentivise proficiency.
3. Schedule monthly section‐specific staff meetings to ensure knowledge transfer across nodes.

Microscopy is a scientific discipline, and the field of ‘microscopy sciences’ requires dedicated, tailored training programs. Investing in these structured training cycles ensures that despite evolving technologies and shortening academic timelines, the global microscopy community continues to produce reproducible, high‐impact science that stands the test of time.

By integrating a tiered classification system with a hybrid blended‐learning model, we have created a scalable framework that balances high‐volume training with the technical rigour required for advanced microscopy. This approach empowers platform scientists to pivot between roles as instructors and active research collaborators, ensuring that complex data is captured with expert precision while maintaining a manageable staff workload. This adaptable model offers a transferable blueprint for research facilities globally, securing the long‐term reproducibility and impact of microscopy sciences in an increasingly demanding academic landscape.

## AUTHOR CONTRIBUTIONS

Eleanor Kable, Matthew Foley, Vijay Bhatia and Filip Braet contributed equally to the conceptional design and formulation of the manuscript. All authors have reviewed, edited, and approved the final version of the manuscript.

## CONFLICT OF INTEREST STATEMENT

The authors declare that they have no known competing financial interests or personal relationships that could have appeared to influence the work reported in this paper. The authors declare the following financial interests/personal relationships which may be considered as potential competing interests: E.K. reports administrative support was provided by The University of Sydney.

## Supporting information



Supporting Information

## Data Availability

Data sharing not applicable to this article as no datasets were generated or analysed during the current study.
