# Supplementary material for: The changing landscape of training in microscopy facilities: Reflections on seven decades of research user support at the University of Sydney
Source: J Microsc. 2026 May 28;304(3):257–65. doi: 10.1111/jmi.70121 (PMC13740130; doi:10.1111/jmi.70121)
Supplement: Supplementary file 1 [file JMI-304-257-s001.pdf]

Basic Optical Microscope

User's Name: ____

User ID (Unikey): ____

Assessor: ____

Date: ____

Category 2 access grants users the ability to independently reserve and operate this piece of equipment within Category 2 hours (Monday: 12:00 AM – 5:00 PM; Tuesday – Friday: 9:00 AM – 5:00 PM). This status is a privilege contingent upon adherence to the Work Health & Safety (WHS) regulations of the University of Sydney and Sydney Microscopy & Microanalysis (SMM). Non-compliance with these regulations may result in the loss of Category 2 access and potential liability for any damages incurred due to breaches of these guidelines.

- This assessment is specific to the instrument identified above. If I require access to additional instruments within SMM , I must consult the appropriate SMM staff.
- I will not train others unless explicitly authorized by SMM staff, as all training is the responsibility of designated SMM personnel.
- I will not permit unauthorized individuals to access the SMM facility using my swipe card, nor will I leave security doors open upon entering or exiting the unit.
- In the event of an incident, uncertainty regarding appropriate action, or technical issues, I will promptly notify the Duty Microscopist.
- I will respond appropriately to emergencies and comply with all instructions given by SMM staff.
- I will refrain from using the SMM facility if I am unwell, fatigued, or under the influence of alcohol or drugs.
- As a SMM user, I am responsible for adhering to established safety protocols and ensuring that my actions do not compromise the health and safety of others.
- If I have not operated the equipment for a period of six (6) months, I understand that I must complete a reassessment to maintain my Category 2 user status.
- I acknowledge that I have read and understood the associated Risk Assessment (RA) Safe Work Procedure (SWP) for the specified instrument.

By signing below, I confirm that I have reviewed all the statements above and accept the responsibilities and privileges associated with SMM Category 2 User status.

User signature: ____

Competency Checklist

Background Knowledge

Completed all required training sessions.	
Can explain emergency shutdown and evacuation procedures.	
Understands Duty Microscopist system and Cat 2 privileges.	
Understands data storage rules (e.g., USB restrictions, RDS transfer).	
Can identify microscope light paths (BF, EPI, Confocal).	
Understands optical resolution vs. pixel resolution.	
Understands basic effects of intensity, detector gain and offset.	
Prepares samples appropriately for the instrument.	

Start-up

Can turn on the microscope.	
Can mount samples appropriately.	
Can focus on the sample.	
Can perform Kohler Illumination.	
Can change between eye and confocal lightpaths.	

Imaging & Data Storage

Can set up imaging with appropriate OCs.	
Can create, modify, and save OCs.	
Chooses appropriate detector settings.	
Chooses appropriate lasers and laser settings.	
Understands histogram, contrast, and LUT and uses them appropriately.	
Understands the effects of size, scan speed, and pinhole size.	
Can perform multipoint acquisitions.	
Can acquire timelapse images.	
Can acquire z-stacks.	
Can acquire large area scans.	
Can use ND acquisition to combine imaging types.	
Chooses appropriate pixel size (nyquist sampling, size and zoom).	
Can use Transmission Detector.	
Can save images in user folder (on shared data folder).	
Can transfer data to RDS.	

Session Completion

Can safely remove samples and return microscope to standard state.	
Can clean objectives.	
Knows how to log post session comments in FMS.	
Has a storage & disposal plan for samples.	

I agree I have been adequately trained in the items selected above.

User signature: ____

Assessor signature: ____

Complex Optical Microscope

User's Name: ______________________________

User ID ______________________________

Assessor: ______________________________

Date: ______________________________

Category 2 access grants users the ability to independently reserve and operate this piece of equipment within Category 2 hours (Monday: 12:00 AM – 5:00 PM; Tuesday – Friday: 9:00 AM – 5:00 PM). This status is a privilege contingent upon adherence to the Work Health & Safety (WHS) regulations of the University of Sydney and Sydney Microscopy & Microanalysis (SMM). Non-compliance with these regulations may result in the loss of Category 2 access and potential liability for any damages incurred due to breaches of these guidelines.

- This assessment is specific to the instrument identified above. If I require access to additional instruments within SMM , I must consult the appropriate SMM staff.
- I will not train others unless explicitly authorized by SMM staff, as all training is the responsibility of designated SMM personnel.
- I will not permit unauthorized individuals to access the SMM facility using my swipe card, nor will I leave security doors open upon entering or exiting the unit.
- In the event of an incident, uncertainty regarding appropriate action, or technical issues, I will promptly notify the Duty Microscopist.
- I will respond appropriately to emergencies and comply with all instructions given by SMM staff.
- I will refrain from using the SMM facility if I am unwell, fatigued, or under the influence of alcohol or drugs.
- As a SMM user, I am responsible for adhering to established safety protocols and ensuring that my actions do not compromise the health and safety of others.
- If I have not operated the equipment for a period of six (6) months, I understand that I must complete a reassessment to maintain my Category 2 user status.
- I acknowledge that I have read and understood the associated Risk Assessment (RA) Safe Work Procedure (SWP) for the specified instrument.

By signing below, I confirm that I have reviewed all the statements above and accept the responsibilities and privileges associated with SMM Category 2 User status.

User signature: ______________________________

Competency Checklist

Background Knowledge

Completed all required training sessions.	
Can explain emergency shutdown and evacuation procedures.	
Understands Duty Microscopist system and Cat 2 privileges.	
Understands data storage rules (e.g., USB restrictions, RDS transfer).	
Can identify microscope light paths (BF, EPI, Confocal, STED).	
Understands microscope resolution vs. pixel resolution.	
Understands basic effects of intensity, exposure time, detector gain and offset.	
Prepares samples appropriately for the instrument.	

Start-up

Can turn on the microscope.	
Can mount stage insert and samples appropriately.	
Can focus on the sample.	
Can perform Kohler Illumination.	
Can change between eye/camera.	
Can turn on lasers.	
Can choose appropriate bit depth for detectors.	

Imaging & Data Storage

Can set up scan using Dye Assistant or manually.	
Chooses appropriate detector settings.	
Chooses appropriate lasers and laser settings.	
Can identify the different lasers (Argon, WLL, STED, etc).	
Understands histogram, contrast, and LUT and uses them appropriately.	
Understands the effects of format, scan speed, and pinhole size.	
Chooses appropriate pixel size (Nyquist sampling, format and zoom).	
Can take images & select appropriate scan settings.	
Can set up multiple sequences.	
Understands pinhole and how to adjust it.	
Understands averaging vs accumulation and when to use.	
Can use Transmission Detector.	
Can use gating.	
Understands the difference between HyD and PMT detectors.	
Understands the function of the motorised correction collar, and can use it safely.	
Can set up and collect Z-stack.	
Can set up and collect time series.	
Can save images in user folder (on shared data folder).	
Can transfer data to RDS.	

Session Completion

Safely removes samples and resets microscope to default state.	
Cleans objectives, microscope and bench properly.	
Checks next session and logs session comments on FMS.	
Follows sample storage/disposal protocols.	

Experiment-specific Requirements

Live Cell

Knows start up order.	
Can adjust temp and CO_2.	
Can install live cell chamber.	
Can mount sample on the stage.	
Can disassemble live cell chamber.	
Can decontaminate live cell chamber.	

Navigator

Can use Navigator without objective hitting sample.	
Can set up ROIs.	
Can use Focus Map.	
Can use AFC.	
Can merge images.	
Can use with z-stack.	
Can adjust overlap.	

STED

Can launch control software in STED configuration and turn on STED lasers correctly.	
Can set up a STED acquisition.	
Understands the difference between 2D and 3D STED.	
Can optimise STED settings.	
Understands which STED laser to use for a given dye.	
Knows which objectives are appropriate for STED.	
Aware that not all dyes are equally suitable for STED.	
Understands which detectors are appropriate for STED.	

Spectral Scans

Can set up excitation scan.	
Can set up emission scan.	
Can set up lambda lambda scan.	
Can export whole image and ROI intensity data.	
Understands reflection and how to avoid it.	
Can add new dye to dye assistant from lambda lambda scan.	

I agree I have been adequately trained in the items selected above.

User signature: ____

Assessor signature: ____

Instrument Specific requirements

		Date Approved	By Assessor
General Operation			
Live Cell			
Navigator			
STED			
Spectral Scans			
